# Correlation study of male semen parameters and embryo aneuploidy in preimplantation genetic testing for aneuploidy

**DOI:** 10.3389/fendo.2022.1072176

**Published:** 2023-01-26

**Authors:** Hongyi Yang, Yan Liu, Wenbin Niu, Zilan Yang, Yang Wang, Haixia Jin, Gang Li

**Affiliations:** ^1^ Reproductive Medical Center, The First Affiliated Hospital of Zhengzhou University, Zhengzhou, Henan, China; ^2^ School of Basic Medical Sciences, Zhengzhou University, Zhengzhou, Henan, China

**Keywords:** semen parameters, embryo aneuploidy, PGT-A, sperm morphology, sperm DNA fragmentation index

## Abstract

**Objective:**

The purpose of this study was to evaluate the influence of abnormal semen parameters on embryo aneuploidy based on single nucleotide polymorphism microarray (SNP array).

**Methods:**

A total of 464 blastocysts from 103 PGT-A cycles were analyzed. The embryo quality and embryo aneuploidy rates were compared between different groups which divided by male semen parameters (sperm concentration, motility, morphology, and DFI) according the WHO criteria (2021).

**Results:**

The total blastocysts chromosome aneuploidy rate was 42.3% (191/452). In the teratozoospermia group, the good-quality embryo and blastocyst formation rate were lower than the normal group(44.4% vs 60.7%, P <0.01; 33.3% vs 43.5%, P <0.05), The good-quality embryo rate in normal DFI group was significantly higher than high-DFI group (59.0% vs 48.4%, P < 0.05). The blastocyst aneuploidy rate in low sperm concentration group, and high DFI group was no differences between with that in normal sperm concentration and DFI group (47.7% vs 37.8% and 44.7% vs 37.8%, P>0.05). The aneuploid rate of blastocyst in teratozoospermic and asthenozoospermia group was significantly higher than that of normal morphology and motility group (50.0% vs 34.0% and 46.7% vs 33.7%, P<0.05).

**Conclusion:**

Our study revealed that sperm DFI were positively correlated with blastocyst aneuploidy rate, while sperm motility and sperm morphology rate were negatively correlated with blastocyst aneuploidy rate. Abnormal semen parameters may affect embryo quality and increase the aneuploidy rate of blastocyst chromosomes, suggesting that in clinical practice of assisted reproduction patients with abnormal semen parameters can be treated in advance to improve sperm quality, so as to reduce the impact on embryo quality and achieve a better pregnancy outcome.

## Introduction

In Assisted Reproductive Technology(ART)treatment, the embryo quality is a key factor for pregnancy outcomes. Studies have shown that the aneuploidy rate in human early embryos is 56-84% during *in vitro* fertilization (IVF) (1). Aneuploidy embryos not only affects embryo implantation and causes pregnancy failure but can also lead to adverse pregnancy outcomes, such as embryonic abortion and miscarriage during early pregnancy (2). The mechanism of embryonic aneuploidy is complex. Most studies have focused on the influence of female factors (oocyte mitosis errors, female advanced age etc.) on embryo aneuploidy (3, 4). Maternal aneuploidy increases exponentially with female age, reaching up to 80% by age 45 (5). While, there was no association between paternal age and aneuploidy rates (6, 7). The male gamete makes up half of the genetic makeup of the embryo; therefore, male factors also play an essential role in embryo chromosomes. Besides age, male sperms are vulnerable to many factors, such as oxidative stress, that result in damage to sperm DNA or increase the number of aneuploid sperms (8, 9), thereby affecting the embryo quality and pregnancy outcomes. Semen parameter is one of the important indicators for male fertility evaluation, which is closely related to embryo development and pregnancy outcome in ART (10). Burrello et al. (11) found that patients with oligo-astheno-teratozoospermia have an increased aneuploidy rate. However, Rossella et al. (12) showed male factor just impairs early embryonic competence in terms of fertilization rate. The euploidy rate and implantation potential of the obtained blastocysts are independent from sperm quality. Studies have shown that a high sperm DNA fragmentation index (DFI) of sperm increases the early miscarriage rate (13, 14), and embryonic chromosomal abnormality is one of the main causes of early pregnancy loss. A recent meta-analysis also showed that couples with unexplained recurrent miscarriage had significantly increased levels of sperm DFI and significantly decreased levels of total motility compared with couples without recurrent miscarriage (15). Therefore, we hypothesized that male factors are also closely related to embryo aneuploidy.

Preimplantation Genetic Testing for Aneuploidy (PGT-A) is an effective method for detecting embryonic chromosomal abnormalities and screening euploid embryos for transfer. In this study, we retrospectively analyzed embryo quality and blastocyst aneuploidy rates with different male semen parameters (sperm concentration, motility, morphology, and DFI) during PGT-A cycles performed at our hospital to investigate the relationship between male semen parameters with the embryo quality and blastocyst aneuploidy rate in assisted reproductive technology.

## Materials and methods

### Participants and study design

We collected data from 103 PGT-A cycles performed between April 2018 and February 2019 at the Reproductive Center, First Affiliated Hospital of Zhengzhou University. A total of 464 blastocysts were sent for a single nucleotide polymorphism (SNP) array chromosome aneuploidy assay. The inclusion criteria of the cases in this study were: 1) maternal ageless than 35year old; 2) a history of repeated miscarriage or repeated implantation failure; 3) no hereditary disease or chromosomal abnormality detected in either partner. This study was approved by the Ethics Committee of Zhengzhou University.

The male sperm concentration, motility, morphology, and DFI were rated as normal or abnormal according to the World Health Organization (WHO) criteria, 6th Edition (16), as follows: sperm concentration: oligospermia (sperm concentration < 16 x 10^6^/mL) and normal density (sperm concentration ≥ 16 × 10^6^/mL); sperm motility: asthenospermia (sperm motility < 42%) and normal motility (sperm motility ≥ 42%); sperm morphology: teratozoospermia (sperm cells with normal morphology < 4%) and normal morphology (sperm cells with normal morphology ≥ 4%); and sperm DFI: high DFI (DFI ≥ 15%) and normal DFI (DFI < 15%). In order to avoid research bias, in the grouping comparison of semen parameters, only the grouping parameters were different, and other parameters were normal. The rates of day 3 good-quality embryos, blastocyst formation, implantation and blastocyst aneuploidy were compared between groups.

### Semen analysis, sperm morphology assessment and sperm DNA fragmentation assay

All cases in this study were subjected to at least two semen assessments. The men were instructed to abstain from sex for 2-7 days and to collect sperm samples into a sterile container by masturbation. After the semen was completely liquefied, 10 µL of liquefied semen was placed in the sperm Makler counting chamber (Sefi Medical Instruments, Haifa, Israel) for motility and concentration analysis. Hematoxylin and eosin (HE) staining was performed to analyze the sperm morphology according to the Kruger criteria, and the lower limit for normal sperm morphology is 4%. All semen analysis was performed in accordance with the 6th WHO Laboratory Manual for Examination and Processing (2021). Sperm DNA fragmentation was evaluated by DNA fragmentation index (DFI) with the sperm chromatin structure assay (SCSA™). An appropriate amount of semen was diluted in TNE buffer (0.01M Tris-HCL, 0.15M NaCl, 1 mM EDTA) to adjust the semen density to 0.5-1.0 x 10^6^/mL Acridine orange (AO; PH6.0) solution was added for staining, and then the sperm DFI was calculated with fluorescence signals detected with a flow cytometer (BD FACS Canto II). The detailed operation steps refer to the previous description (17).

### Ovarian stimulation, fertilization, and blastocyst biopsy

A long luteal phase protocol was used for controlled ovarian hyperovulation. Oocytes were harvested36-38 hours after the doses of 10000 IU human chorionic gonadotropin (HCG) trigger and fertilized with intracytoplasmic sperm injection (ICSI). The embryos were cultured and observed for development. A blastocyst biopsy was performed on day 5 or 6 after the blastocysts were formed, and 3-5 trophectoderm (TE) cells were collected for genetic testing. After biopsy, all blastocysts were vitrified (Cryotop device and solution, Kitazato) and stored in liquid nitrogen. The euploid blastocysts were transfered during the frozen embryo transfer(FET) cycle. The good-quality embryos rate defined as the number of embryos with Peter grade I or II at the 8-cell stage (day 3 after fertilization)/two pronuclei (2PN) cleavage embryos. According Peter (18), embryos on the third day of development and with six to eight blastomeres, with < 10% of fragmentation, and without multi-nucleation were considered good quality embryos. Blastocyst formation and scoring was followed by Gardner DK, on day 5 or day 6 of development. The embryo develops into a blastocyst with inner cell mass (ICM) and trophoblast cells (TE), and then it can performe a blastocyst scoring according to the Gardner scoring system (19). Good quality blastocyst was defined as having an ICM and TE type A or B.

### Whole genome amplification and blastocysts chromosome aneuploidy definition

After biopsy, the ectodermal trophoblasts cells were collected and placed in 5μl of 0.2 N KOH for cell lysis. Then whole-genome amplification (WGA) was performed with the REPL-g Single Cell Kit (QIAGEN, 150345) according to the package insert. The SNP array data were detected using a Human CytoSNP-12DNA microarray array (Illumina, San Diego, CA, USA) and an Illumina HiScanSQ BeadArray Reader. Chromosome aneuploidy in the blastocysts was analyzed with GenomeStudio Software v2011 (Illumina). The specific steps of this method were described previously (20). The aneuploidy rate was defined as the ratio of the number of blastocysts with abnormal chromosome and the total number of blastocysts subjected to SNP array detection.

### Statistical analysis

SPSS v19.0 was used for the statistical analysis. The data were expressed as the mean ± standard deviation (x ± s). T-tests were applied in the comparison of basic parameters (female age, BMI, AMH level, male age et al.). Before the t-test, statistical software was used to conduct the normal distribution test and homogeneity analysis of variances (Levence-test) for all the data of each group. And nonparametric test was applied when t test requirements were not met. The good-quality embryos rate, blastocyst formation rate, implantation rate and aneuploidy rate were analyzed with the χ2 test. It was considered significant difference when P < 0.05. Binary logistic regression analysis was performed with the relevant factors that may affect good-quality embryos, blastocyst formation and blastocyst aneuploidy rate as independent variables and P < 0.05 was considered statistically significant.

## Results

A total of 464 blastocysts obtained from103 PGT-A cycles received diagnostic by SNP-array karyomap chip. The mean age of men and women were 30.3 ± 5.5 and 32.1 ± 5.7 years. The woman’s average BMI and AMH level were 23.7 ± 4.0 (kg/m^2^) and 3.5 ± 2.7(ng/ml). DNA amplification failed for 12 blastocysts and the failure rate was2.6% (12/464). Of the rest452 blastocysts, 191 were chromosome aneuploid. The total blastocysts aneuploidy rate is 42.3% (191/452).

### The basic information in the different sperm morphology and DFI groups

**Figure 1:**
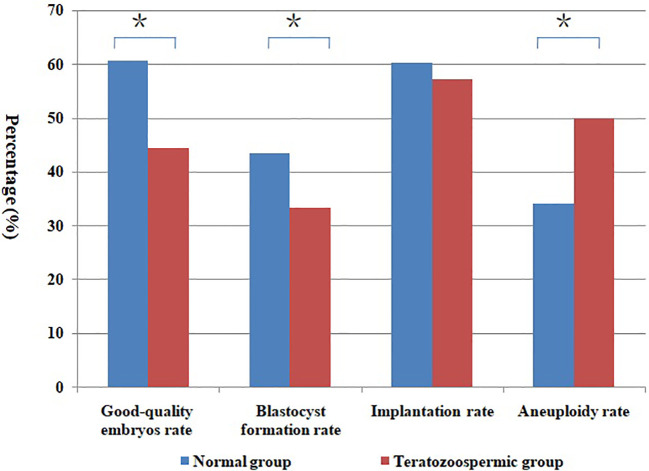
Comparison of different rates between normal and teratozoospermic group. *Statistically significant (P < 0.05).

**Figure 2:**
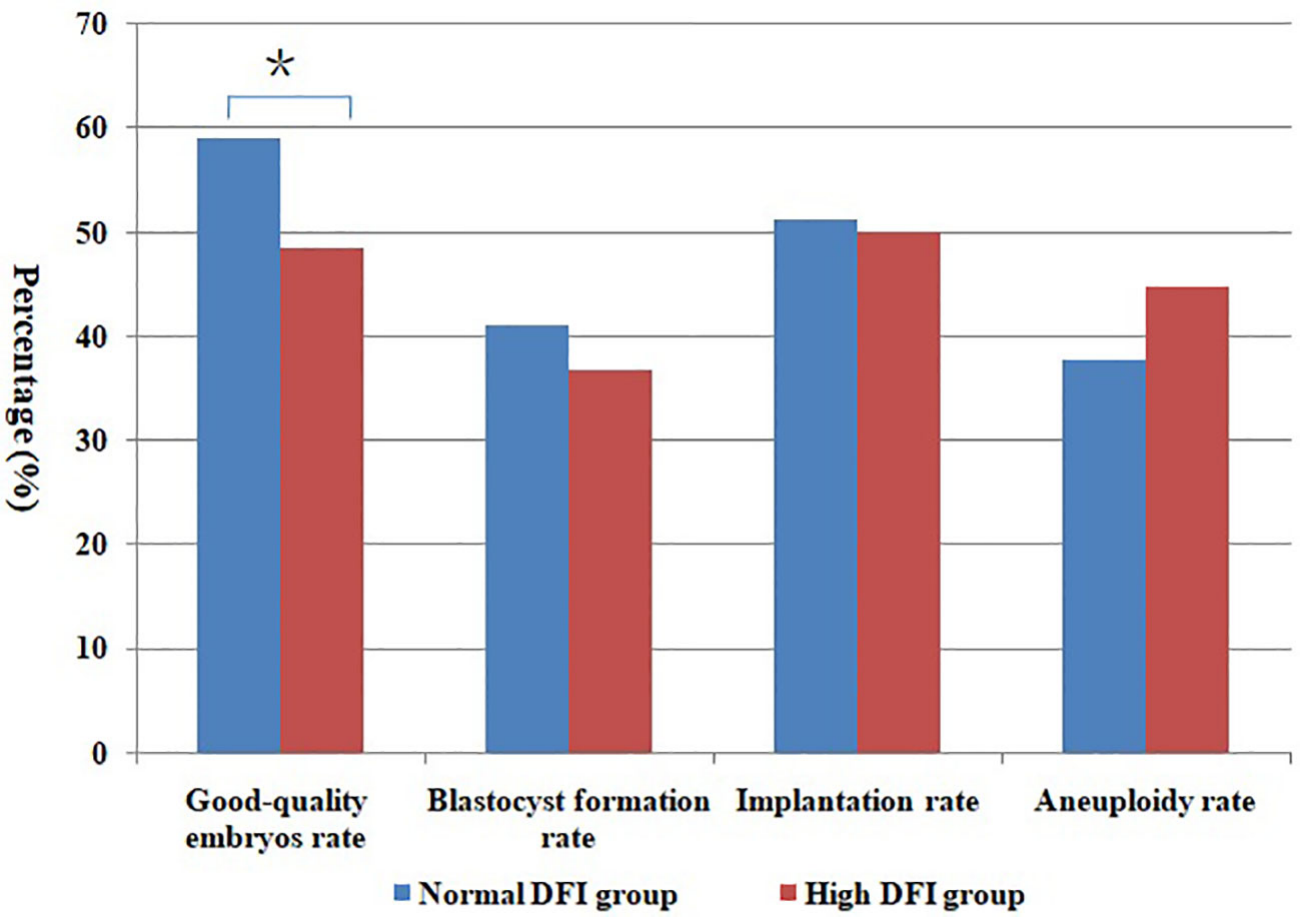
Comparison of different rates between normal DFI and high DFI group. *Statistically significant (P < 0.05).

After the normal distribution and F-test, the basic parameter of female age, BMI, AMH level and male age meet the T-test conditions. Non-parametric test and t- test were used to statistically control other parameters of semen (concentration, motility and DFI) in comparison groups in the tables. And there was no significant difference between groups (P > 0.05). In **Table 1**, the good-quality embryo rate and blastocyst formation rate were 44.4% and 33.3% in the teratozoospermia group, which were significantly lower than the rates of60.7% and 43.5%, respectively, obtained in the normal morphology group (P < 0.05). The blastocyst aneuploidy rate was 50.0% in the teratozoospermia group, which was significantly higher than the rate of 34.0% obtained in the normal morphology group (P < 0.05). Moreover, the good-quality embryo rate was significantly higher in the normal DFI group than in the high-DFI group (59.0%vs48.4%, P = 0.01). But there were no significant differences between two groups in the blastocyst formation rate, implantation rate and blastocyst aneuploidy rate (P = 0.27, 0.93 and 0.28, respectively) (see **Table 2**). **Figures 1**, **2** were clearly shown the comparison of good-quality embryo rates, blastocyst formation rates, implantation rates and chromosome aneuploidy rates in the different sperm morphology and DFI group.

**Table 1:** Comparison of parameters between different sperm morphology groups.

	Normal sperm group	Teratozoospermic group	*P-*value
Cycle (n)	54	14	
Female age (y)	31.1 ± 4.7	29.3 ± 5.5	0.29
Female BMI (kg/m^2^)	23.6 ± 3.9	24.0 ± 4.1	0.45
Female AMH (ng/ml)	3.3 ± 1.8	3.9 ± 2.8	0.51
Male age (y)	32.4 ± 5.2	31.1 ± 5.9	0.39
Male BMI (kg/m^2^)	25.4 ± 3.3	25.8 ± 3.7	0.74
Sperm morphology rate (%)	5.4 ± 0.5	1.8 ± 0.4	<0.01
Sperm DFI (%)	14.1 ± 7.1	22.7 ± 19.7	0.17
Sperm concentration (x10^6^/ml)	53.5 ± 22.3	25.9 ± 21.6	0.22
Sperm motility (%)	42.1 ± 16.7	29.6 ± 17.3	0.34
Good-quality embryos rate	60.7%(357/588)	44.4%(84/189)	<0.01*
Blastocyst formation rate	43.5%(256/588)	33.3%(63/189)	0.01*
Implantation rate	60.3%(38/63)	57.1%(8/14)	0.83
Aneuploidy rate	34.0%(87/256)	50.0%(28/56)	0.02*
Amplification failure rate	3.1%(8/256)	5.4%(3/56)	0.67

*Statistical significant.

**Table 2:** Comparison of parameters between different sperm DFI groups.

	Normal DFI group	High DFI group	*P-*value
Cycle (n)	40	24	
Female age (y)	29.5 ± 5.6	30.7 ± 3.9	0.17
Female BMI (kg/m^2)^	23.4 ± 3.3	23.7 ± 3.5	0.66
Female AMH(ng/ml)	2.9 ± 1.7	3.8 ± 2.6	0.24
Male age (y)	30.7 ± 5.6	33.5 ± 4.4	0.16
Male BMI (kg/m2)	25.4 ± 3.3	25.9 ± 4.0	0.44
Sperm DFI (%)	8.5 ± 4.4	28.5 ± 10.7	<0.01
Sperm morphology rate (%)	5.0 ± 0.6	4.3 ± 0.8	0.29
Sperm concentration (x10^6^/ml)	95.5 ± 32.3	62.5 ± 30.6	0.19
Sperm motility (%)	43.6 ± 10.1	34.8 ± 10.3	0.53
Good-quality embryos rate	59.0%(236/400)	48.4%(124/256)	0.01*
Blastocyst formation rate	41.0%(164/400)	36.7%(94/256)	0.27
Implantation rate	51.2%(22/43)	50.0%(10/20)	0.93
Aneuploidy rate	37.8%(62/164)	44.7%(42/94)	0.28
Amplification failure rate	1.2%(2/164)	4.3%(4/94)	0.26

Except for DFI, other semen parameters between normal DFI and high DFI group were normal, and there was no statistical difference (P>0.05). Good-quality embryos rate in normal DFI group was significantly higher than that of the high-DFI group (P<0.05). * Statistical significant.

### Logistic regression analysis of variables associated with good-quality embryos, blastocyst formation and blastocyst aneuploidy rate

After excluding confounding factors male and female age, sperm concentration and motility, the effects of sperm morphology and DFI on blastocyst euploidy rate, blastocyst formation rate and blastocyst aneuploidy rate were investigated. It can be seen from the **Table 3** that compared with normal morphologic sperm, the good-quality embryo and blastocyst formation rate were decreased in the teratozoospermia group (*P*=0.008 and 0.038), and the risk of blastocyst aneuploidy rate was significantly increased (*P*=0.005).The good-quality embryo rate of high DFI group was lower than that of normal DFI group (*P*=0.031), and the risk of blastocyst aneuploidy rate in high DFI group was 3.213 times higher than that in normal DFI group (OR=3.213, *P*=0.027).

**Table 3:** Logistic regression analysis of variables associated with good-quality embryos, blastocyst formation and blastocyst aneuploidy rate.

Variable	Good-quality embryos rate		Blastocyst formation rate		Blastocyst aneuploidy rae	
	OR (95%IC)	*P*-value	OR (95%IC)	*P*-value	OR (95%IC)	*P*-value
Sperm morphology						
Normal sperm	Referent		Referent		Referent	
Teratozoospermic	0.335(0.271-0.564)	0.008*	0.851(0.692-0.923)	0.038*	6.747(3.743-10.512)	0.005*
Sperm DFI						
Normal DFI	Referent		Referent		Referent	
High DFI	0.696(0.524-0.812)	0.031*	0.913(0.787-1.406)	0.067	3.213(1.772-5.353)	0.027*

*Statistical significant. Logistic regression analysis excluded confounding factors: male and female age, sperm concentration and motility.

### The blastocyst aneuploidy rates with different semen parameters groups

**Figure 3:**
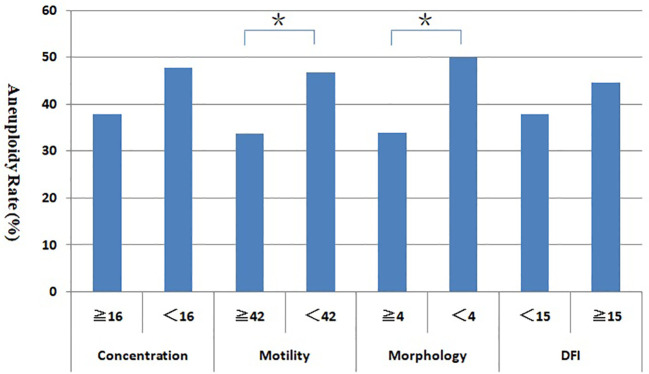
Relationship between sperm parameters and blastocyst aneuploidy rate. *Statistically significant (P < 0.05).

**Figure 3** shows the blastocyst aneuploidy rates with different semen parameters groups (sperm concentration, motility, morphology, and DFI). The aneuploidy rate of blastocyst in abnormal semen parameters group were higher than that in normal group, especially in teratozoospermia and asthenospermia group, the blastocyst aneuploidy rate was significantly higher in the normal group (P < 0.05).

## Discussion

At present, assisted reproductive technology (ART) has become an important way to address infertility. The quality of the embryo is closely related to clinical pregnancy rate in IVF. The high aneuploidy rate of early embryos greatly affects the embryo quality is a major factor for adverse pregnancy outcomes (21). In this study, the blastocyst aneuploidy rate averaged 42.3%, this ratio is similar to the results of Huang’s research (22). This high rate may relate to the population in this study, because the aneuploidy rate of embryonic chromosomal is higher in older women and patients with repeated pregnancy failure than in the general population (23). Garcia et al. conducted a study in a PGT-A population and also observed a high blastocyst aneuploidy rate (24). Generally, human embryo aneuploidy is believed to derive from the oocyte rather than sperm, which may be related to male versus female differences in gamete meiosis (25). However, studies have shown that as part of the embryo’s genetic make-up, the effect of the male sperm nuclear genome can be detected as early as the single cell zygote stage and then throughout 8-cell stage (26). Sperm genomic defects affect centrosome function in the early stage and interfere with the formation of the prokaryotic nucleolus at the zygotic stage, which affects the number and distribution of nucleoli, causes delayed cleavage or increased fragmentation at the cleavage stage, and affects formation of the blastocyst and pregnancy outcomes in the late stage (27). Male factors are known to affect embryonic development, but the extent of this effect is unclear. In this study, we investigated the effect of the male semen quality on the embryo quality (good-quality embryo and blastocyst formation rates) and the blastocyst chromosomal aneuploidy rate during embryonic development and evaluated how male factors affected embryonic development.

Sperm morphology is an important indicator for evaluation of male fertility and is also a key parameter for predicting pregnancy outcomes of assisted reproductive technology (28). Ditzel et al. (29) showed that globozoospermia was associated with a significantly increased sperm chromosomal abnormality rate. Sperm chromosomes are directly involved in the chromosome composition of fertilized oocytes. Sperm with abnormal chromosome can lead to abnormal chromosome in the embryo after fertilization, with severe impacts on embryonic development, implantation, and the health of the offspring. In this study, the embryo aneuploidy rate was significantly higher in the teratozoospermia group (percentage of normal morphology sperms < 4%) than in the normal morphology group, and was also the cause of adverse pregnancy outcomes and high miscarriage rates in the teratozoospermia group. Dubey et al. (30) conducted a study of 52 IVF- PGD cycles and showed that the percentage of sperms with normal morphology was significantly correlated with the clinical pregnancy and implantation rates, which were lower in the teratozoospermia group than in the normal group. Moreover, the rate of embryonic chromosomal abnormalities on day 3 was higher in the teratozoospermia group than in the normal group. Kahraman et al. (31) showed that PGT-A significantly improved the implantation rate in patients with macrocephalic spermatozoa. Thus, performing PGT-A is may be an advantageous option for patients with a relatively low percentage of normal morphology sperms to screen embryos with a normal karyotype for implantation and improve the pregnancy and implantation rates.

The IVF technique bypasses most of the natural selection mechanisms during sperm-oocyte fertilization, which may be one cause of the increased embryonic aneuploidy rate in assisted reproductive technology. In particular, during ICSI, sperms bypass the biological screening that occurs during natural fertilization. The technique to a large extent relies on the subjective evaluation of sperm morphology by embryologists. However, sperm morphology may not reflect chromosomal abnormalities. Studies have shown that the embryo aneuploidy rate is higher in ICSI than in conventional IVF (32). A low percentage of sperms with normal morphology increase the chance of injecting sperms with chromosomal abnormalities or genetic mutations into the cytoplasm of an oocyte during ICSI, resulting in embryonic chromosomal abnormalities, impaired embryonic development, or embryo aneuploidy. In patients with a high percentage of sperms with normal morphology, the chance of selecting sperms with normal morphology is higher during ICSI, which improves the quality of embryos after fertilization and reduces the blastocyst aneuploidy rate. Recently, new assisted reproductive technologies, such as motile sperm organelle morphology examination (MSOME) and intracytoplasmic morphologically selected sperm injection (IMSI), have been introduced in clinical practice with an aim of selecting sperms with normal morphology to improve the quality of embryos and pregnancy outcomes (33). These technologies highlight the important role of sperm morphology in pregnancy outcomes.

Semen parameters are closely related to sperm aneuploidy (34). Studies have shown that the ratio of aneuploid sperms is less than 10% for normal semen but is significantly higher for abnormal semen (35). Aneuploid sperms may result in aneuploid embryos after fertilization. Aneuploidy embryos were significantly higher in couples with abnormal semen parameters (36). Our study showed that the rates of blastocyst aneuploidy was no difference between low sperm concentration group and normal concentration group (47.7% vs 37.8%, P = 0.07). The rate of blastocyst aneuploidy in the low sperm motility group was significantly higher than that in the normal sperm motility group (46.7% vs 33.7%, P<0.05). Dai’s research showed that couples with unexplained RM had significantly decreased levels of total motility compared with couples without recurrent miscarriage, but no differences were observed in the semen volume, sperm concentration, and total sperm count between couples with and without recurrent miscarriage (15). This is exactly consistent with the conclusions of our study. The ICSI procedure reduces the need for sperm count, thus reducing the effect of sperm concentration on embryo development and aneuploidy. However, high sperm motility means that sperm have normal function and must be related to embryonic development and euploidy.

Sperm DFI is an important measure of sperm damage and reflects the integrity of sperm genetic materials. During sperm maturation, the histones that bind to DNA are gradually replaced by protamine. Protamine is connected to the DNA strand *via* disulfide bonds to form a unique dense structure of sperm nuclei (37). The instability of disulfide bonds makes sperms prone to damage during maturation and transport. Oxidative stress, endogenous nuclease abnormalities, protamine deficiency, and apoptotic abnormalities associated with various microenvironmental disorders interfere with the highly ordered series of reactions that occur during sperm formation and maturation, which ultimately lead to DNA damage and infertility. Many studies have shown that a high sperm DFI is associated with a high rate of early miscarriage, whereas embryonic chromosomal abnormalities are an important cause of early pregnancy loss. Therefore, we hypothesized that a high sperm DFI was closely related to embryo aneuploidy. This study showed that the blastocyst aneuploidy rate in the high-DFI group is not significant higher than that in normal DFI group, (44.7%vs37.8%, P = 0.28). This is the same result as Bibi’s study (36). Increase in DFI did not correlate with embryonic aneuploidy, could be to oocytes’ potential to activate mechanism to arrest development of aneuploidy embryos. Researchers are still debating the effect of DFI on pregnancy outcomes in assisted reproductive technology (12, 38). We believe that the method of semen pretreatment may be one of the important factors causing the deviation of results. Density gradient centrifugation and upstream treatment remove most sperms with abnormal morphology and poor motility, thus ensuring the selection of high-quality sperms for ICSI, which reduces the effect of male factors on the embryo quality. Bungum et al. (39) used SCSA to analyze the DNA integrity of sperms selected with density gradient centrifugation and found no significant difference in the sperm DFI between the clinical pregnancy group and the nonpregnancy group undergoing assisted reproductive technology. These results suggest that semen treatment may affect pregnancy outcomes of assisted reproductive technology probably a suitable method of preparation is more effective in removing sperms with potential abnormal morphologies, thereby reducing the embryonic aneuploidy rate.

As a retrospective study, this study has certain limitations. Sample selection bias and the small sample size may affect the accuracy of the results. The sample size is too small. The inclusion criteria of the cases may weaken the reliability and the reason and mechanism are complex or highly variable among different couples. We will try our best to expand the sample size in future studies to exclude the confounding influence of non-research indicators and improve the accuracy of research results. In addition, embryo chromosomal mosaicism may affect the accuracy of the results. In human early embryos, the rate of chromosomal mosaicism is as high as 40% at the cleavage stage and is approximately 10% at the blastocyst stage (40). Embryo mosaicism is very important factor in preimplantation stage. However, in this study, no mosaicism embryos were detected. Firstly, the sample is limited and we performed blastocyst biopsy, which greatly reduced mosaicism rate. On the other hand, a large number of cells were collected during biopsy, which reduced amplification failure, improved the accuracy of the results, and minimized the diagnostic error associated with mosaicism embryos. And most importantly, the detection technique we used (Human CytoSNP-12DNA array from Illumina) is indeed limited in its ability to detect mosaicism embryos, which can only be detected a very high percentage of mosaicism. Our previous strategy was to attribute this high proportion of mosaicism directly to chromosomal abnormalities, so this is the main reason for the absence of mosaicism data in this study and this is also an important limitation of our study. In future studies, we will use NGS, which is more sensitive to embryo mosaicism detection, in order to obtain more accurate results.

## Conclusions

This study revealed that high sperm DFI was negatively correlated with good-quality embryo rate and positively correlated with blastocyst aneuploidy rate, while sperm motility and sperm morphology rate were negatively correlated with blastocyst aneuploidy rate. Abnormal semen parameters may affect embryo quality and increase the aneuploidy rate of blastocyst chromosomes, suggesting that in clinical practice of assisted reproduction patients with abnormal semen parameters can be treated in advance to improve sperm quality, so as to reduce the impact on embryo quality and achieve a better pregnancy outcome.

## Data availability statement

The original contributions presented in the study are publicly available. This data can be found here: https://doi.org/10.6084/m9.figshare.21922695.v1.

## Ethics statement

This study was approved by the Ethics Committee of the First Affiliated Hospital of Zhengzhou University. The patients/participants provided their written informed consent to participate in this study.

## Author contributions

Conceived and designed the experiments: GL, HY. Performed the experiments: HY, YL. Collected the data: WN, YW, HJ. Analyzed the data: ZY. Wrote the manuscript: HY. Proofread the manuscript: GL. All authors reviewed and approved this manuscript. All authors contributed to the article and approved the submitted version.
